# Increased circulating follicular helper T cells with decreased programmed death-1 in chronic renal allograft rejection

**DOI:** 10.1186/s12882-015-0172-8

**Published:** 2015-11-03

**Authors:** Jian Shi, Fengbao Luo, Qianqian Shi, Xianlin Xu, Xiaozhou He, Ying Xia

**Affiliations:** Third Clinical College of Soochow University, Changzhou, Jiangsu China; The University of Texas Medical School at Houston, Houston, TX USA

**Keywords:** Chronic renal allograft rejection, Tfh cells, PD-1

## Abstract

**Background:**

Chronic antibody-mediated rejection is a major issue that affects long-term renal allograft survival. Since follicular helper T (Tfh) cells promote the development of antigen-specific B cells in alloimmune responses, we investigated the potential roles of Tfh cells, B cells and their alloimmune-regulating molecules in the pathogenesis of chronic renal allograft rejection in this study.

**Methods:**

The frequency of Tfh, B cells and the levels of their alloimmune-regulating molecules including chemokine receptor type 5 (CXCR5), inducible T cell co-stimulator (ICOS), programmed death-1 (PD-1), ICOSL, PDL-1 and interleukin-21 (IL-21), of peripheral blood were comparatively measured in 42 primary renal allograft recipients within 1–3 years after transplantation. Among them, 24 patients had definite chronic rejection, while other 18 patients had normal renal function.

**Results:**

Tfh-cell ratio was significantly increased with PD-1 down-regulation in the patients with chronic renal allograft rejection, while B cells and the alloimmune-regulating molecules studied did not show any appreciable change in parallel.

**Conclusions:**

The patients with chronic renal allograft rejection have a characteristic increase in circulating Tfh cells with a decrease in PD-1 expression. These pathological changes may be a therapeutic target for the treatment of chronic renal allograft rejection and can be useful as a clinical index for monitoring conditions of renal transplant.

## Background

Renal transplantation remains an effective treatment for end-stage renal dysfunction [1], facilitating a return to normal health and prolonging life. However, antibody-mediated rejection is a major issue that affects long-term renal allograft survival. Despite the rapid development of new immunosuppressive drugs for attenuating acute rejection, improving the long-term survival of grafts is still a challenge mainly because of chronic allograft rejection. The features of chronic renal allograft rejection are hypertension, proteinuria, progressive deterioration of graft function, peritubular capillary C4d deposition, presence of donor-specific antibodies (DSA) and morphological changes with transplant vasculopathy, glomerulopathy, fibrosis and lymphocyte infiltration. However, the causes leading to chronic rejection are complex and not well understood yet [2].

Allograft rejection is characterized by an increase in activated CD4+ T-lymphocytes, especially regulatory and cytotoxic T cells, leading to an imbalance of immune responses in the transplant recipients [3, 4]. Functionally, CD4+ T helper cells that interact with antigen-specific B cells are required for the production of alloantibodies [5]. Among them, Tfh cells, a recently defined subset of CD4+ T cells, play a particular role in mediating B cell-driven allogeneic responses. Tfh cells can migrate into germinal centers and promote B-cell activation and differentiation into immunoglobulin-producing plasmablasts or plasma cells [5]. They can express PD-1, CXCR5, ICOS, IL-21 and the transcription factor B-cell lymphoma 6 (Bcl-6) [6, 7], thereby displaying their regulatory functions.

Circulating Tfh cells, peripheral counterparts of conventional Tfh cells, express PD-1, CXCR5, ICOS and IL-21, but not Bcl-6 [5–7]. They play an important role in human humoral immunity through these functional molecules. Their abnormal activities are critically involved in the onset of several human diseases such as autoimmune disorders, cancer and infective diseases [7–10]. Therefore, an alteration in circulating Tfh cells may be correlated with disease conditions and might be used as a biomarker of certain diseases [11–13]. Moreover, recent clinical studies have shown that peripheral Tfh cells in the kidney transplant recipients with acute rejection can regulate B-cell alloreactivity and the number of these Tfh cells alters the immunization status and DSA levels [14]. However, their function and relevance to chronic renal allograft rejection are not known yet.

This study was conducted to explore the potential association between circulating Tfh cells and chronic rejection in kidney transplant recipients. The outcome results may provide a useful hint for clinical prediction of renal status after transplantation and for a potential new therapy for chronic allograft rejection.

## Methods

This study was approved by the Institutional Ethics Committee of Third Affiliated Hospital of Soochow University, Jiangsu Province, China. Written-informed consent was obtained from all participants of the study.

### Subjects

The patients with primary renal transplantation for 1–3 years were enrolled from October 2013 to December 2014. Totally 42 recipients were studied in this work, including 24 patients with chronic rejection (CR group) and 18 patients with normal renal function as the normal control (NC group). All of them received the treatment with cyclosporine A, methylprednisolone and mycophenolate mofetil or azathioprine after the renal transplantation. The diagnosis for chronic allograft rejection was confirmed by renal biopsy, biochemical measurements and immunological assays, including DSA as described in other studies [15–18]. In specific,the diagnostic criteria included 1) clinical evidence of slowly deteriorating graft function; 2) biopsy evidence and diffuse deposition of C4d; and 3) the presence of circulating DSA at the time of biopsy. Their peripheral blood samples were collected in a standard way by the clinical laboratory of the hospital. All the subjects had no infective disease when sampling blood.

### Surface staining and flow cytometry analysis

The whole blood was subjected to flow cytometry detection by a BD bioscience FACSCantoII cytometer with FACSDiva software for measuring the frequency of circulating Tfh cells and B cells as well as the expression of their surface markers. The following conjugated monoclonal antibodies were used to stain the cells: CD4-FITC, CXCR5-APC, ICOS-PE, PD-1-PE, CD19-PE-Cy5.5, ICOSL-PE and PDL-1-APC. The cells were incubated with the antibodies for 30 min at room temperature in the dark. Totally 50,000 lymphocytes were acquired in each sample. Data were analyzed using Flow Jo software 7.6.1.

### Enzyme-linked immunosorbent assay (ELISA)

The levels of serum IL-21 were quantified by using the human IL-21 ELISA kit (eBioscience) according to the manufacturer’s instructions. The concentration in each individual sample was calculated according to the standard curve.

### Statistical analyses

All experimental data were analyzed by Graph Prism version 5.0. The results were expressed as mean ± SD and subjected to *t* test for statistical comparisons between the NC and CR groups. If a *p*-value was found to be less than 0.05, the result would be considered statistically significant.

## Results

### Demographics

The general information of the renal transplant recipients was summarized in Table 1. Gender and age were similar between CR and NC groups. The mean serum creatinine (sCr) and blood urea nitrogen (BUN) were almost three-fold higher in the CR patients than those of NC group (*p* < 0.001).

**Table 1:** General information of the renal transplant patients

Group	Gender	Age	SCr	BUN
	(male/female)		(μmol/L)	(mmol/L)
NC	16/8	42 ± 7	95.4 ± 18.6	5.3 ± 1.6
CR	12/6	49 ± 9	277.0 ± 124.3***	15.2 ± 7.8***

*NC*, normal control; *CR*, chronic rejection. *P* < 0.001 *** vs. NC

### Increased circulating Tfh cells in the CR patients

**Fig. 1:**
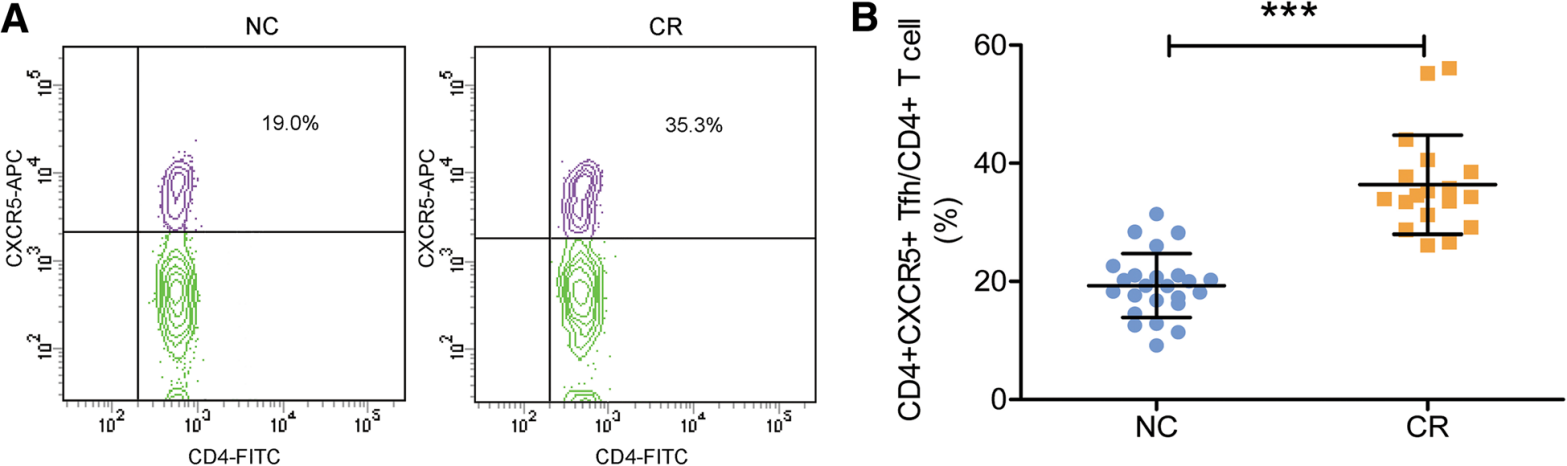
Frequency of Tfh cells in the patients with renal allograft. **a**, representative contour plots of the ratio of Tfh cells in the NC and CR groups, **b**, mean values of the frequency of Tfh cells in the two groups. ***, *P* < 0.001. Note a significant increase in Tfh cells in the CR group compared to that of the control group

To determine if chronic rejection was associated with an alteration in circulating Tfh cells in the renal transplantation recipients, we first evaluated the frequency of CD4 + CXCR5+ Tfh cells through flow cytometry. As shown in Fig. 1, the percentage of CD4 + CXCR5+ Tfh cells among total CD4+ T cells was significantly increased in the CR group as compared to that of the NC group (35.3 ± 8.5 % vs.19.0 ± 5.0, *P* < 0.001).

### Differential changes in PD-1, CXCR5, ICOS, and IL-21 of Tfh cells in the CR patients

**Fig. 2:**
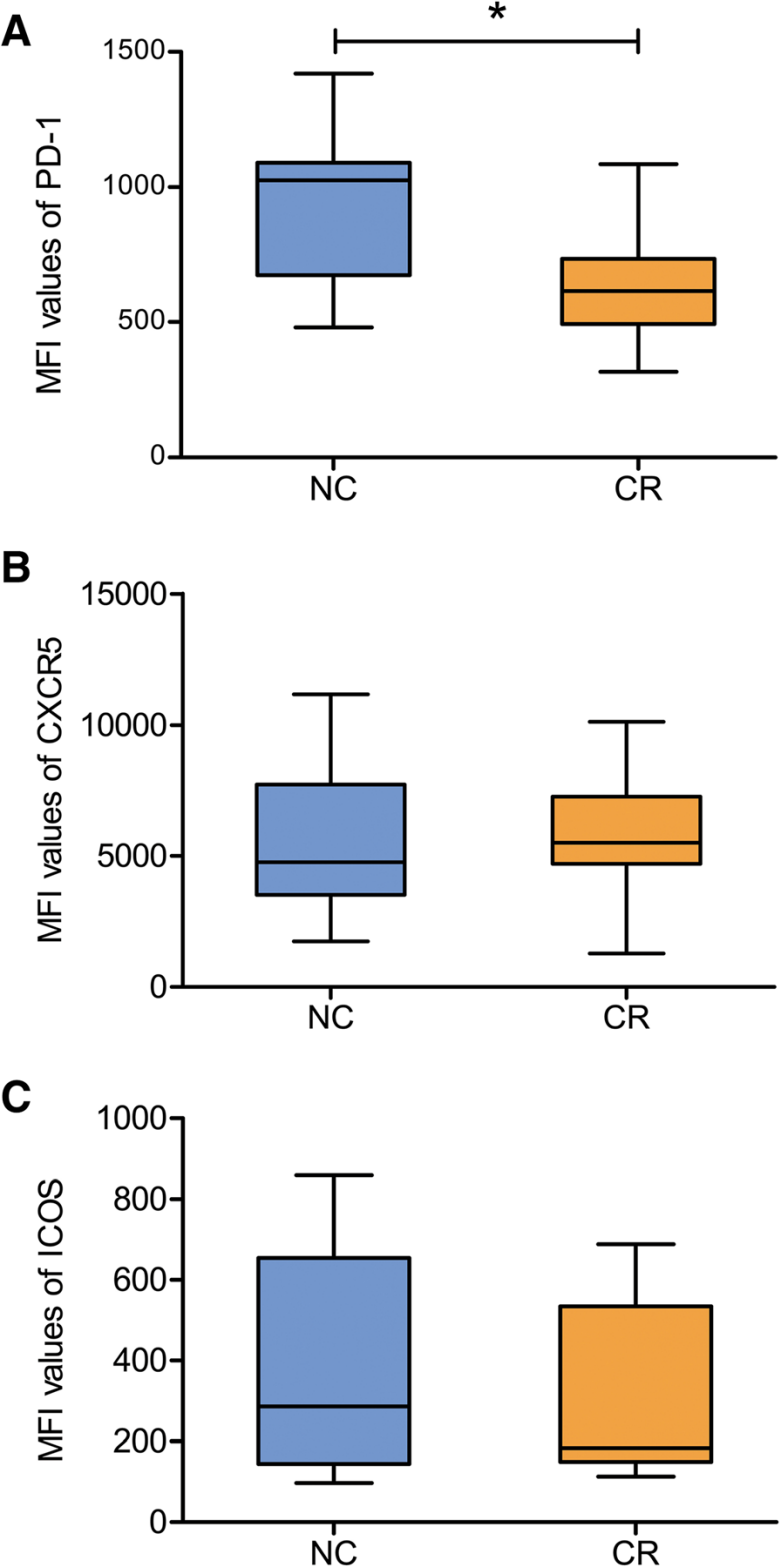
Comparative changes in PD-1, CXCR5 and ICOS expression on Tfh-cell surfaces. **a**,PD-1. **b**, CXCR5. **c**, ICOS. * *P* < 0.05. Note a significant decrease in PD-1 expression with no appreciable changes in CXCR5 and ICOS expression in the CR patients as compared to the NC group

We further detected the changes of PD-1, CXCR5 and ICOS in the high frequency of Tfh cells and found that PD-1 was significantly down-regulated in these Tfh cells (Fig. 2a). In sharp contrast, there were no significant changes in CXCR5 and ICOS in the CR group as compared to the NC group (Fig. 2b and c).

**Fig. 3:**
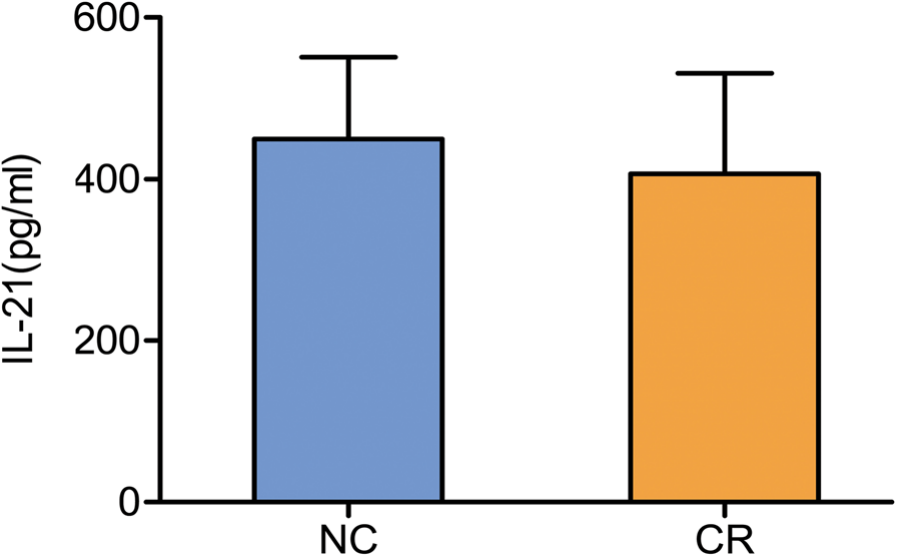
The level of serum IL-21 in renal allograft patients. Note that there was no statistic difference in the IL-21 level between the CR and NC groups

IL-21 is a biological hallmark of Tfh cells because this immune cytokine is produced by Tfh cells and is involved in mediating Tfh-B-cell interaction [6]. Therefore, we further measured the concentration of IL-21 in the serum of the CR patients (Fig. 3). Similarly as the changes in CXCR5 and ICOS, serum IL-21 did not show any significant change in the CR patients as compared to that of the control group (406.9 ± 123.9 pg/ml vs. 449.1 ± 101.7 pg/ml, *P* > 0.05).

### No changes in circulating B cells, PDL-1 and ICOSL in the CR patients

**Fig. 4:**
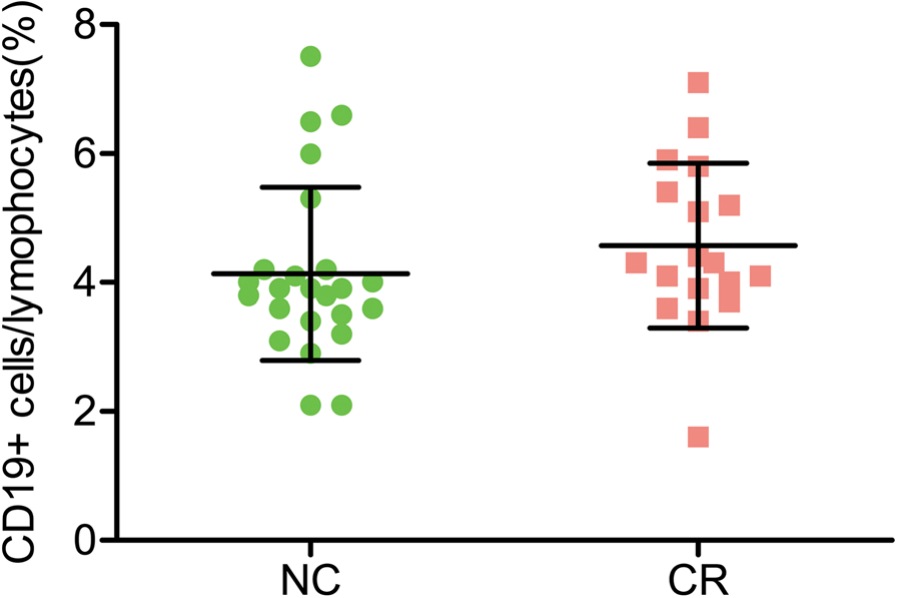
The frequency of B cells. Note that there was no significant difference between two groups

**Fig. 5:**
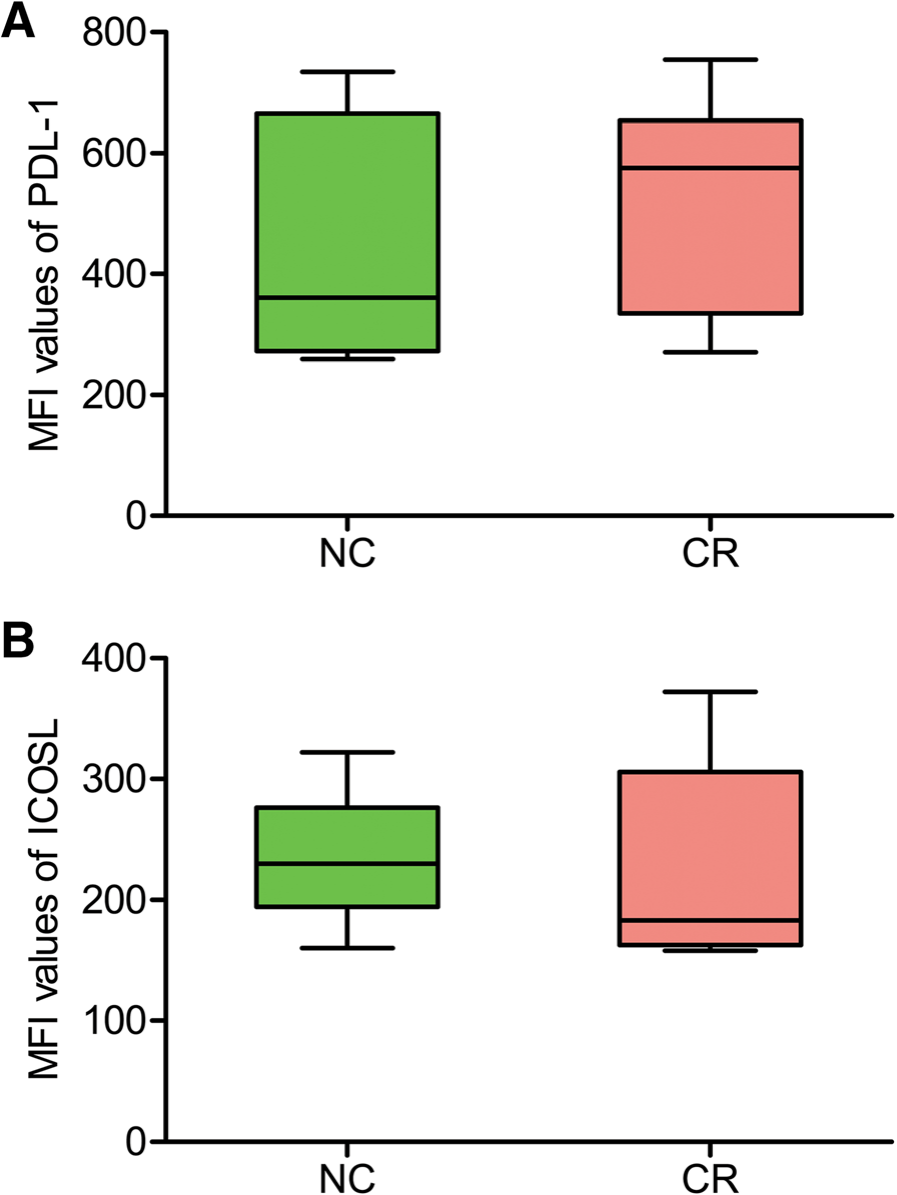
The expression of PDL-1 and ICOSL in B cells. Note that there were no appreciable differences in both PDL-1 (**a**) and ICOSL (**b**) between two groups

Since B cells are key players in the graft rejection [19], we further determined the frequency of total B cells and compared their change with that of Tfh cells. The ratio of B lymphocytes was not significantly different between CR and NC groups (Fig. 4), unlike Tfh cells. Because PDL-1-expressing B cells interact with PD-1+ Tfh cells to regulate the maturation and survival of B cells [20], we next detected the expression of PDL-1 in B cells. Unlike the change in the PD-1 of Tfh cells, PDL-1 did not decrease in B cells at all in the CR group (Fig. 5a). Also, ICOSL had no significant change in the CR patients (Fig. 5b).

All these results suggest that the increase in circulating Tfh cells with PD-1 down-regulation is a specific and characteristic change in the CR patients.

## Discussion

We have made a novel finding in this work, i.e., a major increase in circulating Tfh cells with a significant decrease in PD-1 in the patients with chronic renal allograft rejection. In sharp contrast, B cells and the alloimmune-regulating molecules such as CXCR5, ICOS, ICOSL, PDL-1 and IL-21 did not show any appreciable change in parallel.

Tfh cells display multiple features for their helper functions in secondary lymphoid organs or tissues with inflammation [21]. They migrate into B cell follicles of germinal centers [22] and thereby help B cells generate antibodies for humoral immunity [3, 6, 23]. In fact, Tfh cells, as an immune regulator, are critically involved in the pathological processes of many immune diseases [8]. In renal allograft rejection, the germinal center reactions are dependent on Tfh, while B cells are indispensable for the immune attack to the newly transplanted kidney [24].

Tfh cells express PD-1 [6], while B cells produce PDL-1, an endogenous ligand of PD-1 [19]. The PD-1/PDL-1 signaling has been shown to play an important role in regulating immune functions and affecting the activation of regulatory T cells, cytotoxic T cells and Dendritic cells [25]. Such signaling also influences the generation and differentiation of Tfh and B cells themselves [26]. Recent evidence suggests that blocking the PD-1 signaling induces an up-regulation of Tfh generation and differentiation, which may directly lead to autoimmune encephalomyelitis [27]. In contrast, stimulating this pathway can prolong the survival of the patients after cardiac allograft transplantation [28]. More recently, PD-1 ligands are found to protect the kidneys from ischemia reperfusion injury [29]. In fact, PDL-1, the endogenous ligand of PD-1, has been demonstrated as a required factor for peripheral transplantation tolerance and protection aganist chronic allograft rejection [30].

Taken together, PD-1 signaling is a key regulator for attenuating the Tfh cells and down-regulating the overreaction of humoral immunity against the transplanted kidneys. Therefore, the novel finding of the present study strongly suggests that the deficiency of PD-1 expression causes the increase in Tfh cells, thereby leading to an overreaction of humoral immunity against the allergenic organ, which may be a major reason for chronic allograft rejection.

Tfh and B cells also express many other immune-regulating molecules such as CXCR5, ICOS, ICOSL, and IL-21 [6, 19, 26]. All these molecules are actively involved in the regulation of immune function [26]. For example, an increase in the expression of CXCR5 can enable Tfh cells to migrate into germinal centers [21]. On the other hand, ICOS, another surface receptor like PD-1, also mediate the generation, development and function of Tfh cells by activating ICOS/ICOSL signaling [31, 32]. Moreover, IL-21,a pro-inflammatory cytokine secreted by Tfh cells,has an important role in Tfh cell differentiation, B cell proliferation [33], and the expression of PD-1 [34] and CXCR5 [26]. However, all of these immune regulators did not shown any change in the patients with chronic allograft rejection. We are therefore confident that the deficient PD-1 expression with increased circulating Tfh cells is a specific and characteristic change in chronic allograft rejection.

In the lymph nodes, CD4+ CXCR5+ Tfh cells are more effective in helping B cells than their peripheral counterparts [7]. Humoral response in the lymph nodes can be suppressed by anti-CD40 mAb via regulating Tfh cells [23]. In the transplanted kidneys with acute rejection, infiltrated Tfh cells have been found to participate in the antibody-mediated rejection [14]. However, little is known about the role of these special Tfh cells in the transplanted kidneys with chronic rejection. We speculate that the increase in circulating Tfh cells with a decrease in PD-1 expression might, at least partially, contributes to the genesis of the renal chronic rejection by migrating and infiltrating into germinal centers of renal allografts and lymphoid organs. We will further clarify this issue in our future work.

In addition, pre-existent DSA storing before renal transplantation and *de-novo* DSA developing after renal transplantation are associated with antibody-mediated rejection and allograft failure [35]. However, recent studies have shown that despite the numbers of circulating Tfh cells were higher in the patients with pre-existent DSA than those without pre-existent DSA, the levels of circulating Tfh cells were not different among the patients with or without *de-novo* DSA [35, 36]. Therefore, the relationship between the frequency of circulating Tfh cells and the level of DSA in renal allograft rejection is not clear yet and needs more investigations.

## Conclusions

Our first data show that decreased PD-1 expression may contribute to the increase in circulating Tfh cells in the patients with chronic renal allograft rejection. This finding provides a potential hint for a new target for the treatment of chronic rejection. Moreover, a dynamic change in the expression of PD-1 and the number of circulating Tfh cells may be used as an index for monitoring chronic allograft rejection after kidney transplantation as.

## Abbreviations

Tfh: Follicular helper T cells
CXCR5: Chemokine receptor type 5
ICOS: Inducible T cell co-stimulator
PD-1: Programmed death-1
ICOSL: Inducible T cell co-stimulator ligand
PDL-1: Programmed death-1 ligand
IL-21: Interleukin-21
Bcl-6: Transcription factor B-cell lymphoma 6
CR: Chronic rejection
NC: Normal control
ELISA: Enzyme-linked immunosorbent assay
sCr: Serum creatinine
BUN: Blood urea nitrogen
DSA: Donor-specific antibodies.
